# Outside in

**DOI:** 10.1371/journal.pgen.1008014

**Published:** 2019-02-28

**Authors:** Jonathan Flint

**Affiliations:** Center for Neurobehavioral Genetics, University of California Los Angeles, Los Angeles, California, United States of America

Understanding the relationship between genes and behavior remains a major challenge, and whilst we geneticists have become better at identifying the loci that contribute to behavioral variation (at least in our own species), progress from locus to biological insight leaves a lot to be desired. Most people agree that to turn a robustly identified locus into a gene involves fine-mapping, identifying likely causative variants, finding out the tissue and cell type in which they act, and so on. Missing from this line of reasoning is clarity about what to do once the gene is found: perhaps we should detect enrichment in functional annotations, engineer a mutation in a mouse, or ask a neuroscientist what to do?

To a large extent, the problem is of the geneticist’s making, since the mapping experiment was never designed with the intention of providing a handle on how to understand the neurophysiology of the phenotype. For example, if we want to find the genetic basis of anxiety, our main concern is that our assay captures what we want it to capture, namely anxiety. That seems, to geneticists, the obvious way to proceed. Why would we want the phenotypic assay to capture anything else? After all, the strength of the genetic approach is the unbiased nature of the screen. We certainly don’t ask the phenotype to help us understand its biological origins. We expect the results of the genetic analysis to do that. In brief, we proceed from inside the phenotype and move out towards its genetic constituents (an “inside out” approach).

Neuroscientists think differently. For example, in attempting to understand the biology of anxiety, they look for a specific stimulus, ideally something like a chemical, find the receptor for that stimulus, the neuronal pathway that leads from the peripheral receptor to the central nervous system and delineate the circuitry involved. I’ll call this the “outside in” approach. The idea is that once we have in hand the specific molecular stimulus, identified the receptor and the afferent pathway, we are well on the way to creating a laboratory system, *in vitro*, that recapitulates the elements of the behavior, from external to internal. To give an example, Eric Kandel showed that in the California sea slug (Aplysia californica) natural sensory stimuli (the wave falling on the animal’s skin), could be replaced by a chemical stimulus (serotonin) and the response (gill withdrawal) could be replaced by a recording electrode that detects an action potential from a neuron innervating muscle [1]. In other words, Kandel had found a laboratory preparation, a neuronal circuit in a dish, which modeled the learning processes characterized by behavioral psychologists in the first part of the 20^th^ century. Of course this strategy is hard with some phenotypes (memory for example), but when it does work it offers a chance of uncovering molecular mechanisms in their entirety.

It turns out that ‘outside in’ could serve the geneticists’ needs as well as the neuroscientists, at least in those cases where the peripheral signal elicits a hardwired (for which read ‘genetically-determined’) behavior. Hardwired behaviors are relatively well documented in many species, thanks to Karl Lorentz and Niko Tinbergen. Examples include nesting geese who, at the sight of an egg outside their nest, will extend a bill to gently roll the egg back to inside [2]. If the egg is removed as the goose begins to extend her neck, she still completes the pattern of rolling the non-existent egg back to the nest. What Tinbergen referred to as the bird’s fixed action pattern can be triggered by anything outside the nest that even marginally resembles an egg (beer cans and baseballs do well).

Goose genomics is currently an under-developed field of research. The Avian Phylogenomics Project (aiming to sequence 48 genomes about 10,500 bird species by 2020) published 48 avian genomes in 2014 [3], without including a goose. A pink-footed goose genome was published in 2018 [4], but sadly that’s not sufficient to really break open the genetics of fixed action patterns. Fortunately there are mouse behaviors reminiscent of Tinbergen’s fixed-action patterns, in fact the idea has been around for a while that a subset of sensory neurons in mice is genetically hardwired to gain access to the central neural circuits of aggression [5], and the genomic/connectomic tools and resources for mice are as good as it gets. One example of a mouse hard-wired behavior that has been subject to molecular dissection is the avoidance and risk assessment that chemicals from snakes, cats and rats induce. Lisa Stowers identified major urinary proteins as the stimulus, which activate the vomeronasal organ (VNO) [6].

The VNO is a chemoreceptor organ, separated from the nasal cavity in most amphibia, reptiles and nonprimate mammals [7], which detects pheromones. The VNO system has a number of features that, unlike its anatomical neighbour the olfactory system, suggest the VNO serves as a hotline to behavior, presumably activating subsets of neurons that have a high probability of generating pre-wired behaviors. Activity in VNO neurons has been linked to mating, male aggression, parenting behavior and male territorial urine-marking [8–10].

VNO and olfactory systems are not only anatomically separate, their receptors also function differently. Each olfactory neuron expresses a single sensory G-protein coupled receptor, while VNO neurons express more than one receptor [11]. The upstream pathways into the brain are distinct: olfactory neurons project to the olfactory bulb while the signal from the VNO proceeds to the accessory olfactory bulb, and then splits to feed the medial amygdala and bed nucleus of the stria terminalis. All of this augurs well, indicating a system dedicated to fast-tracking behavioral responses, and providing an experimentally tractable model for teasing apart the relationship between gene and behavior.

Unfortunately, the closer we’ve got to understanding how the system works, the murkier things have become. The division between olfactory and VNO systems isn’t clear cut: deletion of the trace amino acid receptor TAAR4 in the olfactory epithelium converts the response from innately aversive to attractive [12], indicating that some of the olfactory neurons have a more fixed relationship with behavior than first appreciated, and questioning the need for an anatomically separate VNO. Work from Lisa Stowers and Catherine Dulac exposes the VNO system in detail, revealing that the conversion of pheromones into behavioral response is not always consistent with a pre-determined fixed-action pattern. For example two major urinary proteins (MUP3 and MUP20) act in a combinatorial fashion to elicit aggression, and can also elicit urinary countermarking, depending on the context [9]. Dulac found what she termed a ‘multi-sensory logic’ in the way inputs from the VNO were integrated with visual and other information to determine whether a virgin male mouse commits infanticide or displays parenting skills towards a recently born pup [13]. Here, remarkably, one of the pheromones turned out to be haemoglobin (which I guess is unlikely to be marketed, in the way that has happened to oxytocin, as a spray for men to make them more parental). The idea of action-specific stimuli is becoming harder to sustain. But it certainly isn’t dead, as work from another species demonstrates.

There is one recent observation that gave me pause for thought. In another experiment using the ‘outside in’ approach, starting with pheromones, Vanessa Ruta examined how two species of *Drosophila* (*melanogaster* and *simulans*) confer opposite behavioral responses to the same molecule (7,11-heptacosadiene). Explanations for species-specific behaviors surely involve genes. Indeed, it is hard to think of any example where behavior is more likely to be genetically encoded than in the comparison of two, relatively closely related species. In this case the same pheromone promotes courtship in *D*. *melanogaster* males, and suppresses courtship in *D*. *simulans* males. What could be a better target for a genetic explanation of behavioral differences?

The assumption, tacitly made in almost every piece of writing on the subject, is that the complexity of neuronal wiring required to generate a behavior will result in its conservation through speciation, with changes in behavior attributed to alterations in the peripheral sensory receptors. According to this hypothesis, to make a pheromone elicit a different behavior it should only be necessary to change the receptor, or the receptor’s response. The circuitry controlling mating is assumed to be modular in nature, with internal components assigned to separable input and output streams. But that’s not how it is. In fact the receptors remain the same; it’s the circuitry that changes to elicit the opposite responses [14]. Ruta describes a change in the balance of excitatory and inhibitory signalling to convert the pheromone signal, which gives clues as to what to look for as genetic determinants of the change, and, potentially therefore, a handle on the genes involved.

What genes might be involved? The circuit is intact, so it’s probably not the usual developmental culprits that establish and maintain connectivity. Rather we are more likely looking at the effect of neuromodulators that tune the system in one direction or another. After all, as Eve Marder has pointed out, under the heel of neuromodulators the same circuit can be forced to do completely different things [15]. If so, then we could at last begin to assign a role to those neuropeptides that have so far played a bit part in neuroscience, forgotten during the rush to assemble connectomes, which are expected to uncover a neuronal code set to rival that of DNA’s in its explanatory capacity. Of course, whether the lesson from Drosophila neurobiology applies also to mammalian circuits is not known. Indeed any attempts to draw comparisons between invertebrates and vertebrates soon falls foul of our ignorance as to whether organizational principals are sufficiently well conserved to transverse species boundaries.

I’d argue that where it can be applied (and I accept it won’t work for every behavior) “outside in” approaches could replace genetic screens. They are worth considering because of the poor performance of genetic screens in identifying the biological mechanisms underlying behavior. ‘Outside in’ approaches are becoming increasingly tractable, due to the ease with which genomes can be sequenced, and the ease of with which they can be manipulated with CRISPR technologies. But, as this brief tale illustrates, the key is not just having the right technology, it is finding the right species to study. We should not be limited by researching only within the short list of approved model organisms: goose genomics might be a good career choice for a new investigator.
